# Aryl Hydrocarbon Receptor: A New Player of Pathogenesis and Therapy in Cardiovascular Diseases

**DOI:** 10.1155/2018/6058784

**Published:** 2018-06-10

**Authors:** Tao Yi, Jinxia Wang, Kaixi Zhu, Yaoliang Tang, Shian Huang, Xiaorong Shui, Yuanlin Ding, Can Chen, Wei Lei

**Affiliations:** ^1^Laboratory of Cardiovascular Diseases, Guangdong Medical University, Zhanjiang 524001, China; ^2^Cardiovascular Medicine Center, Affiliated Hospital of Guangdong Medical University, Zhanjiang 524001, China; ^3^Vascular Biology Center, Department of Medicine, Medical College of Georgia, Augusta University, Augusta, GA, USA; ^4^Laboratory of Vascular Surgery, Guangdong Medical College, Zhanjiang 524001, China; ^5^Institute of Medical Systems Biology, Guangdong Medical University, Dongguan 523808, China

## Abstract

The aryl hydrocarbon receptor (AhR) is a DNA binding protein that acts as a nuclear receptor mediating xenobiotic metabolism and environmental responses. Owing to the evolutionary conservation of this gene and its widespread expression in the immune and circulatory systems, AhR has for many years been almost exclusively studied by the pharmacological/toxicological field for its role in contaminant toxicity. More recently, the functions of AhR in environmental adaption have been examined in the context of the occurrence, development, and therapy of cardiovascular diseases. Increasing evidence suggests that AhR is involved in maintaining homeostasis or in triggering pathogenesis by modulating the biological responses of critical cell types in the cardiovascular system. Here, we describe the structure, distribution, and ligands of AhR and the AhR signaling pathway and review the impact of AhR on cardiovascular physiology. We also discuss the potential contribution of AhR as a new potential factor in the targeted treatment of cardiovascular diseases.

## 1. Introduction

Aryl hydrocarbon receptor (AhR) is a ligand-activated transcriptional factor belonging to the superfamily of basic helix-loop-helix/Per-ARNT-Sim (bHLH/PAS) [1], and is the only member of this family known to bind naturally occurring xenobiotics [2]. Traditionally AhR acts as a crucial regulator mediating xenobiotic metabolism and environmental responses, and it was discovered to bind closely with 2,3,7,8-tetrachlorodibenzo-p-dioxin (TCDD, dioxin) and then was hyperactivated to release a myriad of toxicologic outcomes, which contribute to the potency of TCDD as an inducer or promoter of some carcinogenesis in 1982 [3, 4]. Thus, for many years, AhR has been almost exclusively studied by the toxicological field for its role in various environmental and food contaminants such as polycyclic aromatic hydrocarbons, polychlorinated biphenyls and dioxins. Numerous studies have found that cytosolic AhR can be activated by many natural and synthetic ligands, and translocated into the nucleus where it complexes with the AhR nuclear translocator (ARNT) [5]. The complex recognizes the specific dioxin-responsive elements (DREs) and modulates subsequent transcription of its downstream target genes including phase I and phase II metabolic enzymes, which can affect the metabolism of environmental toxicants and chemical substances [6].

The increasing deterioration of the natural environment is having serious consequences on human health. The circulation system is the major organ exposed xenobiotics and endobiotics during metabolic homeostasis [7], and long-term exposure to environmental pollutants can drastically alter this system, resulting in cardiovascular diseases such as hypertension, atherosclerosis, and ischemic heart disease [8–12]. Because many environmental pollutants contain exogenous aryl hydrocarbon receptor (AhR) ligands, increasing attention is being given to the relationship between AhR and cardiovascular diseases. Recent evidence from gene knock-out studies and clinical trials suggests that not only does AhR have a major impact on general physiological functions, including immune responses, reproduction, oxidative stress, tumor promotion, the cell cycle, and proliferation [13, 14], but also influences cardiovascular physiological functions [15–18].

In this review, we discuss the progress of AhR biology and toxicology, its pathophysiology roles in the heart and vascular systems, and the prospects as a therapeutic target for cardiovascular diseases, with the aim of providing a potential direction for the prevention and treatment of the diseases.

## 2. AhR

### 2.1. The Structure of AhR

**Figure 1:**
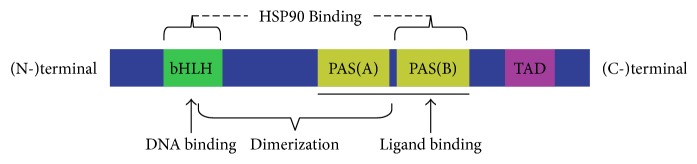
Structure of AhR.

Anthropogenic AhR comprises 848 amino acid residues and has three functional domains, including the bHLH domain, Per-ARNT-Sim (PAS) domains (A and B), and the transactivation domain (TAD), that span from the amino (N-) terminal to the carboxy (C-) terminal [1, 19] (Figure 1). The amino acid sequence of the bHLH domain and the PAS domains are both highly conserved among species [20]. The bHLH domain is located at the (N-) terminal and can divide into an HLH domain and a basic domain, which determines dimerization of the protein molecule and the combination of AhR with DNA [21]. The main role of the PAS A and B domains is to participate in binding to ligands, release of heat shock protein 90, and increase the stability of the heterodimer AhR-ARNT complex to further affect conformation of DNA [1, 22]. The TAD domain functions as a mediator in transcriptional activation of downstream genes [23].

### 2.2. Distribution of AhR in Fetal and Adult Tissues

**Figure 2:**
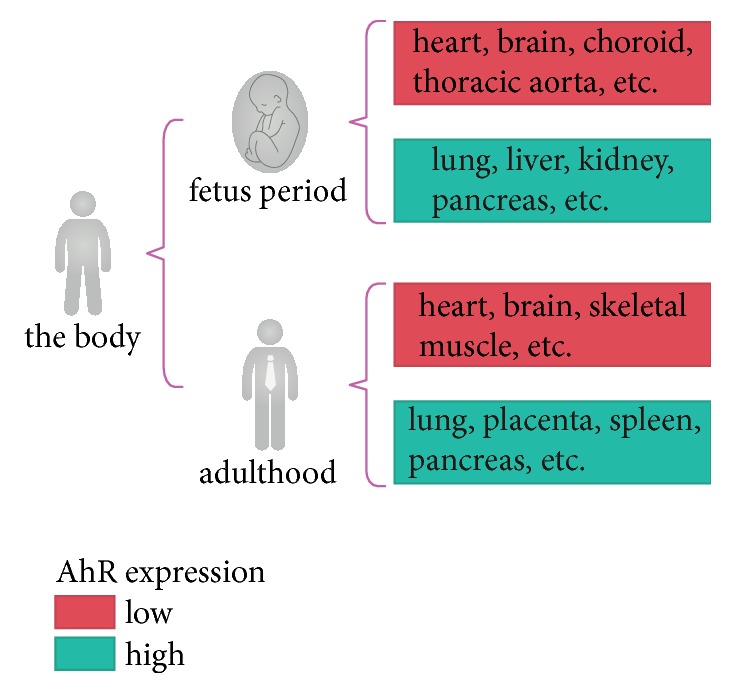
Expression levels of AhR in adult and fetal tissues.

AhR is expressed ubiquitously in the fetus and in adults, with the distribution changing significantly with age [24] (Figure 2). In the fetus, there are specific distribution of AhR in the lungs, liver, kidneys, pancreas, testicles, esophagus, thymus glands, retinas, and epithelial cells, and relatively low levels in the heart, brain, choroids, thoracic aorta, and sclera; In adults, AhR is expressed at relatively high levels in the lungs, placenta, spleen, pancreas, and liver, and relatively low levels in the heart, brain, and skeletal muscles [25, 26]. AhR exerts diverse physiological effects depending on where it is located in different tissues.

### 2.3. The Ligands of AhR

**Figure 3:**
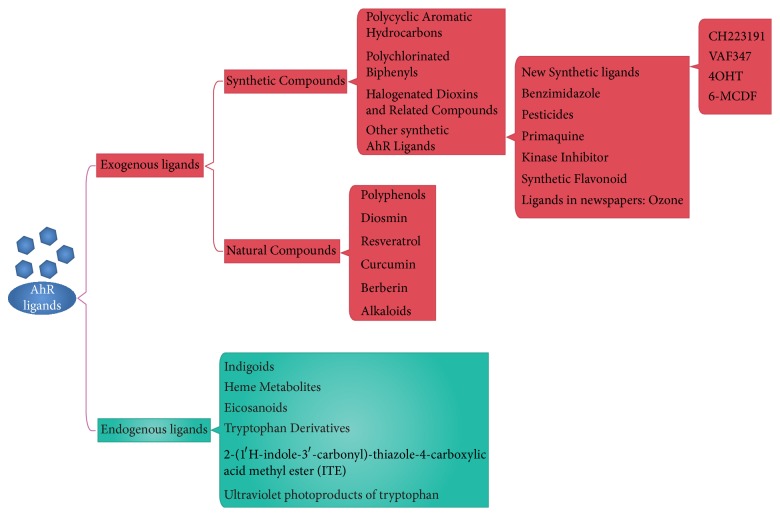
Exogenous and endogenous ligands of AhR.

AhR ligands can be divided into endogenous ligands and exogenous ligands (Figure 3). Endogenous ligands include indigoids, heme metabolites, eicosanoids, tryptophan derivatives, and equilenin [27]. Exogenous ligands include polycyclic aromatic hydrocarbons, polychlorinated biphenyls, natural compounds, and small molecule compounds [28]. The different structures and properties of AhR ligands mean that when they combine with AhR they have distinct biological effects.

### 2.4. The AhR Signaling Pathway

**Figure 4:**
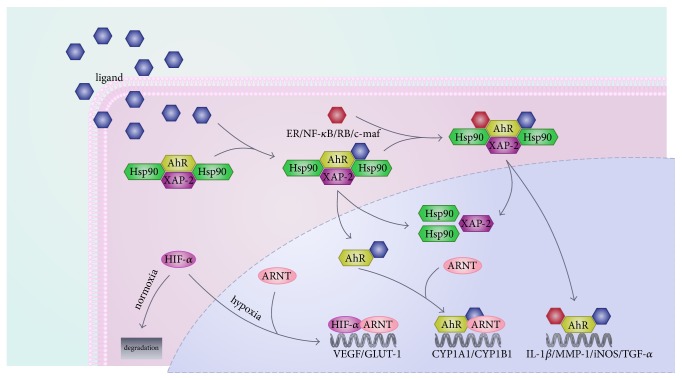
Classical and non-classical AhR signaling pathways.

The AhR signaling pathway involves both classical and non-classical signal transduction mechanisms [2, 24] (Figure 4). In the classical signaling pathway, AhR exists as an AhR molecular chaperone complex comprising an AhR, two heat shock protein 90, and X-associated protein 2 and 23 in the cytosol [24, 29]. Being activated by ligands, AhR is translocated from the cytosol to the nucleus where it disassociates from the complex. The ligand-AhR complex combines with ARNT and binds to a specific DNA promoter sequence called DRE or xenobiotic responsive element (XRE). Ultimately, transcription of a large number of target genes activates and triggers various biological and/or toxicological effects [30].

In the non-classical signaling pathway, the AhR signaling pathway can interact with other pathways by competition for transcriptional coactivators or corepressors [30]. For instance, crosstalk between AhR and hypoxia can interact through competition with ARNT [22]. In the nucleus, sustained AhR activation results in G1 phase cell cycle arrest via hyperphosphorylation of retinoblastoma protein and repression of E2F-dependent transcription [31–35]. In macrophages, AhR, in combination with signal transducer and activator of transcription 1 and nuclear factor-*κ*B (NF-*κ*B), inhibit the promoter activity of interleukin-6 (IL-6) induced by lipopolysaccharide [36]. AhR also regulates the development of regulatory type 1 T cells by combining with the transcription factor c-Mcf [37]. Additionally, numerous studies have described interactions between AhR and estrogen receptors, RelB, RelA, *β*-catenin, and nuclear factor-like 2[38–43].

## 3. Role of AhR in Cardiovascular Physiology

### 3.1. AhR in Cardiac Function and Cardiomyogenesis

Despite low expression levels of AhR in the heart, AhR does have noticeable effects on the physiological functioning of the heart. For example, a study has reported obvious cardiac hypertrophy in AhR−/− mice at 5 months, with increasing levels of the *β*-myosin heavy chain and *β*-myosin light chain 2V. It was suggested that the underlying mechanism may be associated with the elevated level of vascular endothelial growth factor (VEGF) in AhR−/− mice [44]. In 2003, Vasquez et al. observed that cardiac hypertrophy induced in AhR deficiency showed low indices for contractility, pre-load, afterload, cardiac output, stroke volume, and minimal fibrosis, differing from pressure- or volume overload-related cardiac hypertrophy [15]. The researchers suggested that AhR deficiency mainly lead to cardiomyocyte hypertrophy, resulting in cardiomyopathy and cardiac hypertrophy [45]. Paradoxically, another study reported that cardiac hypertrophy in AhR−/− mice was caused by pressure overload and accompanied by evident fibrosis and elevated expression of plasma endothelin-1 (ET-1) and angiotensin II (Ang II). Captopril, an angiotensin-converting enzyme inhibitor, was used to alleviate the pressure overload, leading to a lowered expression of plasma ET-1 and Ang II and a delay in the increase of mean arterial pressure [16]. Subsequent research found that cardiac function in AhR−/− mice could be completely reversed with BQ-123, an ET_A_ receptor antagonist, indicating that ET-1 could be mediated by AhR and function as the key molecule in the progression of cardiac hypertrophy [46]. A recent study revealed that Vav3, an activator of Rho/Rac GTPases, regulated by AhR, was closely associated with cardiac hypertrophy and fibrosis in AhR−/− mice [47]. Nevertheless, the specific mechanism has yet to be completely determined. Although there are contradictions among studies, it is evident that AhR signaling in cardiac function is important.

The AhR signaling pathway is vital for the development of the heart. When AhR was activated by dioxin, transforming growth factor *β* (TGF-*β*)/bone morphogenic protein (BMP) and WNT signaling pathways were disrupted, cardiomyocyte differentiation of enterochromaffin cells stopped, and cardiogenesis was impaired during early differentiation [48]. When AhR was silenced by short hairpin RNA interference in P19 cells, an embryonic carcinoma cell line, expression of the downstream signal molecules of AhR such as ARNT and CYP1A1 and the key molecule in WNT signaling, *β*-catenin, were suppressed, following by the increase in expression of the cardiomyogenesis-specific* GATA4* and* Nkx2.5* genes. These results suggest that AhR mediated the differentiation of P19 mouse embryonic carcinoma cells into cardiomyocytes through the AhR and WNT1 signaling pathways [49]. Another study found that activation, inhibition, or knockdown of AhR all could affect cardiomyocyte differentiation of mouse embryonic stem cells; the cause of which was connected with AhR-relating incongruous expression of genes, including genes encoding homeobox transcription factors and polycomb and trithorax group genes [50]. The expression of AhR in the undifferentiated embryonic stem cells impacts their choice of lineage in differentiation, restricting cardiogenesis and commit to a neuroglia cell fate. With regard to self-renewal of embryonic stem cells, a relatively low level of AhR expression was required for cells to retain their stem cell properties. Han et al. propose that after activation by endogenous ligands AhR participates in the coordination of multiple biological processes which define pluripotency and embryonic development, and AhR can regulate cardiogenesis by modulating the cardiac DNA methylome and the expression of imprinting genes [51]. Hence, cardiomyocyte differentiation is a carefully regulated process in which AhR plays a crucial role.

### 3.2. AhR in the Regulation of Vascular Physiological Functions

Maintenance of the function and structure of blood vessels relies in part on laminar fluid shear stress. Normally, laminar fluid shear stress-activated AhR mediate cell cycle arrest by activating CYP1A1 in human umbilical vein endothelial cells, suggesting the involvement of AhR in the regulation of the vascular microenvironment [52]. A study reported that there exists abnormal vascular structures in the liver, kidneys, and hyaloids in AhR−/− mice [53]. A study of hepatic vascular development revealed that hepatic necrosis and decreased perfusion in the fetal liver was the cause of patent ductus venosus and comparatively smaller livers in adult AhR−/− mice [54]. The results align with that of another study in which mutation of DRE binding sites in AhR affected liver vascular development, suggesting that DNA binding is necessary for AHR-mediated developmental and TCDD-induced toxic signaling [55].

When AhR was activated by 3-methylcholanthrene, an AhR agonist, cell permeability, adhesion, and tube formation of human umbilical vascular endothelial cells was inhibited; but *α*-naphthoflavone, an AhR antagonist, could reverse the effects of 3-methylcholanthrene [56]. Another study reported that TCDD activated the AhR/CYP1A1 and AhR/CYP1B1 pathways, resulting in suppression of angiogenesis, and angiogenic inhibition was reversed with AhR deficiency [57]. Ichihara et al. used middle cerebral artery occlusion in mice and oxygen-glucose deprivation in rat cortical neurons to define the role of AhR in stroke, and the results found that L-kynurenine is an endogenous ligand that mediates AhR activation in the brain, and demonstrated that an L-kynurenine/AhR pathway mediates acute brain ischemic damage after stroke[58]. Ischemia-induced angiogenesis was observed to significantly increase with AhR deficiency, with the effects being associated with the hypoxia-inducible factor-1*α* (HIF-1*α*)-ARNT heterodimer and its downstream gene,* VEGF *[59]. VEGF is necessary for vascularization. Compared with AhR+/+ transgenic adenocarcinoma of the mouse prostate (TRAMP) mice, AhR−/− TRAMP mice showed a higher incidence of prostate cancer accompanied by an increase in VEGF [60]. Similarly, AhR activation inhibited hypoxia-induced VEGF in prostate bone metastasis cells and endothelial progenitor cells, and was associated with HIF-1*α* [61]. It is therefore suggested that AhR may prevent interaction of ARNT with HIF-1*α* and suppress VEGF expression, ultimately blocking angiogenesis.

In summary, AhR participates in the regulation of vascular physiological functions, including vascular development and angiogenesis. Both deficiency and abnormal activation of AhR give rise to vascular dysfunction, and many vascular diseases.

### 3.3. AhR in Blood Pressure Regulation

It is reported that AhR-deficient mice showed decreased cardiac output and low systolic and diastolic aortic pressure compared with normal mice of the same age [15]. In 2010, Zhang et al. reported that the renin–angiotensin system participated in the regulation of normal blood pressure in AhR heterozygous mice but not AhR−/− mice, confirming the importance of the rennin-angiotensin system in the progression of hypotension in AhR−/− mice. ET-1 signaling was also found to be involved in the mediation of hypotension in AhR−/− mice. Importantly, the study reported that the sympathetic nervous system and nitric oxide (NO) signaling were not involved in the activation of the rennin-angiotensin system and ET-1 [62]. AhR−/− mice tend to develop hypertension at a modest altitude (1632 m), caused by hypoxia [63]. Captopril could alleviate high blood pressure in AhR−/− mice, in part because of the reduction of Ang II [16]. Increasing evidence indicates that vascular *α*1D-adrenoceptor overexpression is another influential factor of hypertension in AhR−/− mice, and hypertension could be reversed by treatment with captopril [64]. However, inhibition of ET-1 could not only lower mean arterial pressure and the levels of ET-1, but also reduce Ang II expression levels in AhR−/− mice with hypertension, indicating involvement of the regulation of the ET-1-Ang II axis in hypertension in AhR−/− mice induced by hypoxia [65]. Sauzeau et al. found that AhR controlled cardiovascular and respiratory functions by regulating the expression of the Vav3 proto-oncogene, and demonstrated Vav3 to be a bona fide AhR target in charge of a limited subset of the developmental and physiological functions of cardiorespiratory systems [46]. Taken together, the findings suggest that AhR is involved in the complicated networks that regulate blood pressure, and possible mechanisms should be further studied.

## 4. AhR as a Therapeutic Target in Cardiovascular Diseases

*4.1 AhR and myocarditis. *Myocarditis is a significant cause of heart disease, especially in young people[66]. It can lead to dilated cardiomyopathy, a common precursor of heart failure. Myocarditis can be induced by multiple causes, including infection and auto-immune or auto-inflammatory diseases [67, 68]. Infection remains a major factor in myocarditis and is closely associated with the immune and inflammatory responses of the host. Numerous studies have reported that AhR is a crucial factor in the immune system and is involved in the differentiation of antigen-presenting cells and specific T cell subpopulations [69, 70]. AhR participates in the regulation of innate and adaptive immune responses in some models of infection. AhR is also an important protein to limit the inflammatory response. Deletion of AhR exacerbated the inflammatory response to* Listeria monocytogenes*,* Toxoplasma gondii*, and* Plasmodium falciparum *[71–73], and was confirmed in a model of* Leishmania major *infection [74]. There is strong evidence to suggest that AhR is a pivotal molecule in myocarditis. In 2016, it was first reported that AhR modulated the development of myocarditis during* Trypanosoma cruzi* infection. When model mice were infected with* T. cruzi*, parasitemia, inflammation, and fibrosis of the myocardium were significantly reduced in AhR−/− mice compared with wild-type mice through the reduction in reactive oxygen species (ROS) and cytokines [75]. Viral infection is the most common cause of myocarditis. No study has examined the relationship between AhR and viral myocarditis. However, Coogan et al. found that AhR activation increased the number of neutrophils in the lungs, which contributed to poor survival in mice with influenza virus infection [76]. It is possible that AhR modulates the inflammatory response in viral myocarditis. AhR is a promising line of research on myocarditis.

### 4.1. AhR and Hypertension

It has been reported that exposure to environmental pollutants, particularly traffic-related pollutants, could increase the risk for hypertension [77]; however, causation has not been determined. One possible mechanism is that AhR, as an important regulator of blood pressure, could be activated by abundant exogenous AhR ligands in environment pollutants, such as TCDD [10]. Support for this theory is provided by studies on AhR-/- mice. When AhR was knocked out, mice showed significantly elevated blood pressure as well as elevated Ang II and ET-1 [46]. Another study suggested that 3-methylcholanthrene, an exogenous AhR agonist, can induce high blood pressure associated with endothelial NO synthase (eNOS) inactivation [78]. Endothelial cell-specific AhR-null mice had hypotension, accompanied by increases in eNOS activity and NO production [79]. These findings suggest that AhR could serve as a therapeutic target in hypertension, or other AhR-regulated NO-dependent vascular diseases.

Besides endogenous and exogenous ligands, activation of AhR can also be influenced by genetic polymorphisms. Genetic polymorphisms of the AhR signaling pathway are reported to be closely associated with the pathogenesis of essential hypertension. The majority of single-nucleotide polymorphisms in the AhR pathway, such as rs2228099 (ARNT), rs1048943 (CYP1A1), rs762551 (CYP1A2), and rs1056836 (CYP1B1), are associated with susceptibility to hypertension. The genetic environment and gene–gene interactions in the AhR signaling pathway are reported to determine susceptibility to essential hypertension [80]. Therefore, it is possible that gene therapy targeting AhR signaling could be a potential candidate in the treatment of essential hypertension.

The advent of CRISPR/Cas9, a versatile genome-editing tool, has allowed for precision medicine based on the detection of genetic polymorphisms. CRISPR/Cas9-regulated genome editing is a powerful technology for gene therapy [81]. It is thought that CRISPR/Cas9 will provide great advancements in the potential treatment of hypertension.

It has recently been reported that AhR is expressed in immune cells such as Th17 cells and dendritic cells [70]. Mice lacking T cells exhibited reduced blood pressure increases with Ang II infusion [82]. Whether expression levels of AhR in immune cells influence blood pressure, and possible mechanisms, is an interesting potential area of research.

### 4.2. AhR and Atherosclerosis

Atherosclerosis mainly occurs in the intimal layer of the blood vessel wall, and is characterized by subendothelial lipid deposits, vascular smooth muscle cell migration and proliferation, and formation of foam cells in the subendothelial space [83]. Risk factors for cardiovascular diseases include vascular senescence and obesity. Chronic vascular inflammation and oxidative stress contribute to atherosclerosis [84, 85], but the molecular mechanisms are not well understood. Exposure to contaminants containing ligands of AhR (dioxins, TCDD, PAH, benzo(*α*)pyrene) are thought to promote the development and progression of atherosclerosis, indicating that AhR may play a role in the regulation of atherosclerosis [9, 86–88].

Vascular senescence, a risk factor for cardiovascular diseases, is an important factor in the development of atherosclerosis. Studies suggest that senescent vascular cells are present in human atherosclerotic lesions [89–91]. A study in 2014 reported that indoxyl sulfate regulated sirtuin 1 via AhR activation, promoting endothelial senescence [92]. It was suggested that endothelial senescence in atherosclerosis is linked to AhR activation.

Obesity is also a vital contributor to atherosclerosis. In an AhR-directed luciferase-expressing mouse hepatocyte cell line, treatment with oxidized low-density lipoprotein and transforming growth factor-*β*1 could induce lipid accumulation and luciferase expression, owing to the overexpression of kynurenine, an endogenous AhR ligand, by enhanced indoleamine 2,3-dioxygenase 1 activity. Inhibition of AhR, in turn, prevented obesity [93].

Inflammatory responses contribute to AhR-regulated atherosclerosis. The inflammation-related cytokine monocyte chemoattractant protein-1 (MCP-1), an important endothelium-derived chemokine, was reported to recruit monocytes into the subendothelial space where they differentiated into macrophages, promoting atherosclerotic plaque development [94]. Activation of AhR by TCDD induced the release of a number of inflammatory mediators, including MCP-1, in ApoE-/- mice, leading to the promotion of atherosclerotic lesions and the formation of foam cells [6, 95, 96]. Treatment with CH223191, an AhR antagonist, significantly reduced the development of atherosclerotic lesions induced by TCDD. Expression of MCP-1 triggered by the AhR agonists indoxyl sulfate and coplanar polychlorinated biphenyl 77 could be reversed with the AhR antagonists CH223191 and *α*-naphthoflavone, respectively[94, 97]. ROS also play an important role in AhR-related atherosclerosis. There is evidence to suggest that ROS are involved in the process of indoxyl sulfate-induced MCP-1 production[98]. Indoxyl sulfate activates AhR and promotes ROS production by enhancing NADPH oxidase 4 expression in human umbilical vein endothelial cells. The AhR antagonist, CH223191, could reverse indoxyl sulfate-induced NADPH oxidase 4 expression [99]. A recent report showed that TCDD increased ROS production in endothelial cells and reduced NO-related vasodilation by AhR-dependent pathway which may be mediated, in part, via induction of cytochrome CYP1A1 [88]. Intercellular adhesion molecule-1 and matrix metalloproteinases, both regulated by AhR, may play a role in atherosclerosis [9, 100]; however, the mechanism remains to be determined. Taken together, these studies suggest that AhR could be a potential drug target to interfere with the development and progression of atherosclerosis.

### 4.3. AhR and Ischemic Heart Disease

Ischemic heart disease, including ST-segment elevation myocardial infarction, non-ST-segment elevation myocardial infarction, and stable and unstable angina pectoris, has high global morbidity and mortality. Coronary artery occlusion or stenosis stemming from coronary atherosclerosis is the major cause of ischemic heart disease [12]. Numerous studies have shown that environment pollutants associated with AhR signaling are important factors in atherosclerosis [9, 86–88]. There is evidence to indicate that exposure to exogenous ligands of AhR, such as dioxin, TCDD, and coplanar polychlorinated biphenyls, increase the risk for ischemic heart disease [12]. A study examined the role of AhR in coronary artery disease susceptibility in a Chinese population, and the results suggested that expression of circulating AhR was elevated in patients with coronary artery disease. Further analysis of AhR polymorphisms found that AhR rs2066853 showed a significant correlation with the risk for coronary artery disease [101]. A study at Stanford University reported that the transcription factor TCF21 promoted the expression of inflammation-related genes in human coronary artery smooth muscle cells via interaction with AhR, leading to an increased risk for coronary artery disease [102]. Xue et al. reported that, in myocardial ischemic injury, AhR gave rise to substantial expression of inflammatory cytokines, including high-sensitivity C-reactive protein, interleukin-1*β*, and interleukin-6. However, baicalin, a flavonoid compound, could attenuate the inflammatory response and myocardial injury via suppression of the expression of AhR [103]. Taken together, the findings suggest that AhR may be an important gene or drug target for the prevention and treatment of ischemic heart disease.

### 4.4. AhR and Myocardial Ischemia-Reperfusion Injury

Myocardial ischemia-reperfusion injury is a byproduct of reperfusion after acute myocardial infarction (reperfusion being the best treatment for acute myocardial infarction), and results in cardiomyocyte dysfunction and aggravated myocardial tissue injury [104]. Over the past few decades, researchers have found that ischemic post-conditioning (a treatment other than the traditional ischemic preconditioning) has a cardioprotective effect on ischemic reperfusion injury. In 2013, Vilahur et al. demonstrated that ischemic post-conditioning exerted a protective effect on cardiac structure and function via downregulation of the AhR signaling pathway [105]. Ischemic post-conditioning for myocardial ischemic reperfusion injury could reduce the expression of AhR and ARNT, resulting in a decrease in apoptosis. A recent study found that flavonoids capable of inhibiting AhR have a dual character in myocardial ischemia-reperfusion injury, either protection or deterioration [106]. While there are inconsistencies in the studies, AhR appears to be a significant mediator in myocardial ischemia-reperfusion injury.

### 4.5. AhR and Pulmonary Arterial Hypertension

Pulmonary arterial hypertension, a malignant chronic progressive vascular disease, usually leads to right heart failure and death in the late stage. There are various strategies for the treatment of pulmonary arterial hypertension, such as phosphodiesterase type 5 inhibitor, prostanoids (PGI2), prostacyclin, and endothelin receptor antagonists [107]. However, there is no ideal efficacy among the therapeutic treatments. A new effective therapy is needed, and the role of AhR should be further studied.

A study reported that baicalin, a natural flavone, could attenuate the abnormal proliferation of human pulmonary artery smooth muscle cells induced by TGF-*β*1 via inhibition of the HIF-1*α* and AhR pathways, indicating the participation of the AhR pathway in the progression of pulmonary arterial hypertension [108]. In addition, HIF-1*α* and AhR pathways could interact with each other through ARNT [22]. A study suggested that hypoxic pulmonary hypertension could be attenuated by suppressing HIF-1*α* triggered by hypoxia via RNA interference [109]. Studies examining the effect of the suppression of AhR in combination with suppression of HIF-1*α* in pulmonary arterial hypertension could prove to be useful. Therefore, AhR could be a potential drug target for the treatment of pulmonary arterial hypertension.

## 5. Conclusion

With an increased understanding of the link between environmental pollutants and cardiovascular diseases, the impact of AhR on the cardiovascular system has become evident. AhR plays an important role in maintaining cardiovascular homeostasis. In the cardiovascular system, the absence of AhR can result in abnormal cardiac function, hypertension or hypotension, vascular dysfunction, and cardiovascular disease. The types of cardiovascular disease include myocarditis, hypertension, atherosclerosis, ischemic heart disease, and pulmonary arterial hypertension. The pathogenesis induced by AhR varies among cardiovascular diseases, but includes inflammatory responses, immune responses, oxidative stress, and endothelial dysfunction. The molecular mechanisms behind AhR signaling, crosstalk between AhR signaling and other signaling pathways, and genetic polymorphisms require further study. Genetic polymorphisms of AhR will provide valuable information for guiding targeted gene therapy. Despite progress in our understanding of AhR-relating cardiovascular diseases, crosstalk between the cardiovascular system and the microenvironment is unclear. For targeted medical therapy, an effective dosage is hard to determine. And because most agonists and antagonists of AhR are not tissue-specific, further development is required. Therefore, clinical application of AhR-related therapies still has a long way to go.

AhR is a key bridging molecule in the cardiovascular system. Problems with AhR ligands and AhR transcripts can lead to abnormal activation of AhR, and result in an unbalanced cardiovascular system and cardiovascular diseases. AhR is a potential drug or gene target for the treatment of cardiovascular diseases.
